# Prevalence and Associated Factors of Physical Activity among Medical Students from the Western Balkans

**DOI:** 10.3390/ijerph19137691

**Published:** 2022-06-23

**Authors:** Maja Grujičić, Miloš Ilić, Budimka Novaković, Aleksandra Vrkatić, Zagorka Lozanov-Crvenković

**Affiliations:** 1 Department of General Education Subjects, Faculty of Medicine, University of Novi Sad, Hajduk Veljkova 3, 21000 Novi Sad, Serbia; 2 Department of Pharmacy, Faculty of Medicine, University of Novi Sad, Hajduk Veljkova 3, 21000 Novi Sad, Serbia; milos.ilic@mf.uns.ac.rs (M.I.); budimka.novakovic@mf.uns.ac.rs (B.N.); aleksandra.vrkatic@mf.uns.ac.rs (A.V.); 3 Department of Mathematics and Informatics, Faculty of Sciences, University of Novi Sad, Trg Dositeja Obradovića 3, 21000 Novi Sad, Serbia; zlc@dmi.uns.ac.rs

**Keywords:** physical activity, medical students, young adults, public health, Western Balkans

## Abstract

The student population includes young adults who need nutrition and regular physical activity (PA) for mental, cognitive, and physical development. It is estimated that, globally, only 25–40% of the university student population is involved in regular PA. To date, no research has been conducted in the Western Balkans to address the PA of medical students. The aim of this study was to investigate the prevalence and factors influencing PA among medical students from the Western Balkans. A cross-sectional study included 2452 students from 14 medical faculties in five countries (Slovenia, Croatia, Bosnia and Herzegovina, North Macedonia and Serbia). There were significantly more students who engaged than those who did not engage in some type of regular (daily) PA. Gender, overweight or obesity, and household income are significantly associated with students’ PA. Students who are more often involved in regular daily PA and have higher daily PA levels are more likely to be males whose household income is above average. In order to improve the health of the student population, the public health authorities need to continuously investigate the PA of students and introduce appropriate activities to increase their level of PA.

## 1. Introduction

The World Health Organization (WHO) defines physical activity (PA) as any bodily movement caused by skeletal muscles that requires energy consumption [1]. PA includes all movements even during leisure time, for transport to go to and from places, or as a part of a person’s work [1]. Both moderate- and vigorous-intensity PA improve health [1].

WHO and Centers for Disease Control and Prevention (CDC) global recommendations for PA for the population aged 18 to 64 years emphasize that consistency in the frequency, duration, intensity, type and total amount of daily (regular) PA are necessary to reduce the risk for mass non-communicable diseases (NCD) [2,3]. That means: at least 150 to 300 min of moderate-intensity aerobic PA, or at least 75 to 150 min of vigorous-intensity aerobic PA, or an equivalent combination of moderate-intensity PA and vigorous-intensity PA throughout the week [2,3].

The student population includes young adults whose health habits, i.e., proper nutrition and regular PA are necessary for mental, cognitive, and physical development [4]. When students start attending universities, they usually change their life habits [5]. A high-energy diet high in salt and irregular meals, and a lack of PA can negatively affect student health and lead to overweight and obesity [6,7]. It is estimated that globally 20 to 40% of the university student population is overweight [7,8,9,10]. Overweight and obesity increase the risk of developing NCD (insulin resistance, diabetes, dyslipidemia, hypertension, cardiovascular disease, chronic obstructive pulmonary disease, and other) [11,12]. Regular PA reduces the risk of being overweight and obese, symptoms of depression and anxiety, improves the process of thinking, learning and judgment, and ensures healthy growth and development of young students [2,13]. It also prevents premature mortality caused by NCD [2,13].

Globally, in 2016, 27.5% of all adults was of insufficient PA, with lower activity in women than in men (31.7% vs. 23.4%), which represents great risk for adults due to possible risks of developing or exacerbating diseases connected with inactivity [14]. In 2016, 81% of students population aged 11–17 did not meet current recommendations for daily PA (77.6% of boys and 84.7% of girls), risking their present and future health [15].

The treatable and preventable mortality rate and the percentage of the population that does not spend time on aerobic PA that improves health (unrelated to work) was higher for the Western Balkans countries compared to European Union average (Table 1) [16,17,18,19].

Global research indicates that only 25–40% of university students are involved in regular PA [20,21,22]. Irregular PA is more common in people of better socioeconomic status, people of rural origin, it is associated with alcohol, tobacco and drug abuse, negative social impact (family, peers, social media, etc.), as well as overweight and obesity [4,5,13,20,22,23,24,25].

Physicians and medical students who have healthy life habits are more prone to feel confident in counseling their patients and patients are more likely to trust advice on health behaviors (e.g., diet, PA) given by physicians who lead a heathy lifestyle [26]. Therefore, it is of great significance that medical schools increase the proportion of students maintaining and adopting regular PA habits in order to increase the quality and rates of future physical counseling delivered by physicians [26].

Graduate medical school programs should focus on health promotion in students, because this will probably lead to improved health behavior in students’ patient populations [26].

Up to now, no research has examined PA among the medical students in five countries of the Western Balkans, namely the Republic of Croatia, Republic of Serbia, Republic of Slovenia, Republic of North Macedonia, and Bosnia and Herzegovina.

The objective of this study was to examine the prevalence and factors influencing the PA in medical students from the Western Balkan.

## 2. Materials and Methods

### 2.1. Study Design and Population

A cross-sectional study was carried out in the period from November 2019 to February 2020, among 2452 medical students from 14 medical faculties in five countries of the Western Balkans (Republic of Croatia: Faculty of Pharmacy and Biochemistry of the University of Zagreb and Faculty of Medicine of the University of Rijeka; Republic of Serbia: Faculty of Pharmacy of the University of Belgrade, Faculty of Pharmacy of the University of Business Academy Novi Sad, Faculty of Medicine Novi Sad of the University of Novi Sad; Republic of Slovenia: Faculty of Medicine of the University of Ljubljana; Republic of North Macedonia: Faculty of Medicine and Faculty of Pharmacy of the University “St. Cyril and Methodius” Skopje; and Bosnia and Herzegovina: Faculty of Medicine, Faculty of Pharmacy and Faculty of Health Studies of the University of Sarajevo, Faculty of Medicine of the University of Zenica, Faculty of Pharmacy and Faculty of Health Studies of the University of Mostar). The study was performed by convenience sampling of medical faculties.

### 2.2. Study Questionnaire

The data were collected through an online survey generated on an online platform (Google Form) accessible from any device. The survey was uploaded, and the link was forwarded to the students through student representatives from the home faculty via e-mail and social networks (Facebook), or by posting on faculty websites. The survey participation was voluntary and anonymous. The participants were able to withdraw their participation in the survey at any stage before the submission. When the participants completed the survey linked to the Google Form, each survey was sent to a database from where it could be downloaded as a Microsoft Excel sheet. The online survey was generated so that only the answers from the fully completed survey were registered in the database and were included in further analysis. The privacy of respondents was guaranteed by research method.

### 2.3. Variables

The study was carried out in two parts. The first part included baseline demographic characteristics: gender, faculty the students attend, year of study, body height, body weight, average household income, and type of settlement students lived in before enrolling the university. The second part examined the mean daily PA, alcohol consumption and the smoking status of the respondents.

When asked about the mean daily PA, the respondents checked one of five offered answers: 1. I do not engage in regular PA; 2. Up to 30 min a day; 3. Up to 1 h a day; 4. 1–2 h a day; 5. 3 or more hours a day. The responses: 1–2 h of PA a day and 3 or more hours a day, were categorized as “I engage in PA more than 1 h a day”.

Based on the year of study, medical students were grouped into two categories: 1–3-year students and 4–6-year students.

The body mass index (BMI) was calculated from self-reported data on body height and body weight. The classification was done according to WHO recommendations; underweight: BMI < 18.50 kg/m^2^; normal weight: 18.50–24.99 kg/m^2^; overweight: 25.0–29.99 kg/m^2^; and obesity: ≥30.0 kg/m^2^.

Household income data were gathered by checking one of the five offered answers: 1. Far below average; 2. Below average; 3. Average; 4. Above average; 5. Far above average. Far below average and below average were categorized as “below average household income”, while above average and far above average were categorized as “above average household income”.

In regard to the type of settlement students lived in before enrolling the university, the respondents chose one of the seven offered answers: 1. A village with up to 500 inhabitants; 2. A village from 500 to 3000 inhabitants; 3. A village with over 3000 inhabitants; 4. A town with up to 20,000 inhabitants; 5. A town from 20,000 to 100,000 inhabitants; 6. A town from 100,000 to 1 million inhabitants; 7. A town with over 1 million inhabitants. The responses: a village with up to 500 inhabitants, a village from 500 to 3000 inhabitants and a village with over 3000 inhabitants, were categorized as “rural settlements”, while the responses: a town with up to 20,000 inhabitants, a town from 20,000 to 100,000 inhabitants, a town from 100,000 to 1 million inhabitants and a town with over 1 million inhabitants, were categorized as “urban settlements”.

In order to examine alcohol consumption among the respondents, they were asked to check one of the five offered answers: 1. I do not drink alcohol; 2. Occasionally; 3. On weekends; 4. Several times per week; 5. Daily.

The students were also asked about their smoking habits and the number of cigarettes smoked per day, by choosing one of the six offered answers: 1. I am not a smoker; 2. I smoke occasionally; 3. Up to 5 cigarettes per day; 4. 5–10 cigarettes per day; 5. 11–20 cigarettes per day; 6. More than 20 cigarettes per day. Based on their responses, the students were grouped into “smokers”: I smoke occasionally; up to 5 cigarettes per day; 5–10 cigarettes per day; 11–20 cigarettes per day; and more than 20 cigarettes per day, and “non-smokers”: I am not a smoker.

### 2.4. Statistical Analysis

In statistical analysis, categorical variables were described by frequency distribution and percentages.

A chi-square test was applied to analyze the association between categorical variables, and Cramer’s V was used as association measure.

In order to establish the impact of respondents’ gender, year of study, BMI, alcohol consumption, household income, type of settlement, and smoking status on PA, a binary logistic regression analysis was used with the independent variables coded as follows: gender (0—female, 1—male), year of study (0—1–3, 1—4–6), overweight and obese (0—No, 1—Yes), alcohol consumption (0—I do not drink alcohol, 1—occasionally, on weekends, several times per week and daily), household income (0—below average and average, 1—above average), type of settlement (0—rural, 1—urban), and student smoking status (0—No, 1—Yes). The odds ratio (OR) values were adjusted.

In the binary logistic regression analysis, the dependent variable (student PA) was “Has regular daily PA” (0 = No, 1 = Yes).

Statistical analysis was done using SPSS Statistics for Windows version 24 (IBM Corporation, Armonk, NY, USA). *p* value of < 0.05 was considered statistically significant.

### 2.5. Ethical Aspects of the Research

The study was conducted in accordance with the guidelines of the Declaration of Helsinki, and the Ethics Commissions/Committees of the faculties that took part in the research gave the opinion that the approval of the commissions/committees was not required, since the research did not incorporate invasive methods and violate the privacy of respondents.

## 3. Results

The highest percentage of students was in Bosnia and Herzegovina (35.8%) (Table 2). Among the students, there were more female respondents (82.2%), most presented among students from the Republic of Croatia (88.5%) and least among students from the Republic of North Macedonia (76.1%).

The highest percentage of students were involved in regular PA (62.3%) (Table 3). The daily level of PA differed significantly between different faculties (χ^2^ = 131.882, *p* < 0.001, fi = 0.232). The students from the Faculty of Pharmacy of the University of Mostar, Bosnia and Herzegovina (77.3%), in relation to the students of other faculties, most often engaged in some kind of regular PA, while the highest percentage of students of the Faculty of Pharmacy and Biochemistry of the University of Zagreb, Republic of Croatia (50.9%) did not engage in regular PA.

There was a significant difference in daily level of PA between male and female students only at the Faculty of Medicine Novi Sad of the University of Novi Sad, Republic of Serbia (χ^2^ = 7.957, *p =* 0.047, fi = 0.147) (Table 4). Their female students more often did not engage in regular PA compared to male students (40.2% vs. 28.4%), and male students more often engaged in PA more than 1 h per day in comparison to female students (35.1% vs. 20.3%).

Significant difference in daily level of PA of students in the terms of year of study was determined at the Faculty of Medicine of the University of Ljubljana, Republic of Slovenia (χ^2^ = 9.757, *p* = 0.021, fi = 0.212), where 4–6-year students in higher percentage did not engage in regular PA (52.9%) and had PA up to 30 min a day (21.2%) compared to 1–3-year students (43.0% and 13.2%) and the Faculty of Medicine of the University of Zenica, Bosnia and Herzegovina (χ^2^ = 10.349, *p* = 0.016, fi = 0.225), where 1–3-year students more often did not engage in regular PA (31.5%) compared to 4–6-year students (16.5%) (Table 5).

In comparison with male students, female students more often did not engage in regular PA (39.1% vs. 31.4%) (Table 6). Male students more frequently did have PA up to 30 min (21.1% vs. 19.5%) and more than 1 h a day (27.7% vs. 20.6%) compared to female students, while female students more often had PA up to 1 h per day (20.8%) than male students (19.9%). The difference was significant (χ^2^ = 14.464, *p* = 0.002, fi = 0.077).

Students whose household income was above average significantlly more often engaged in regular PA and had higher level of PA when compared to students whose household income was average and below average (χ^2^ = 19.686, *p* = 0.003, fi = 0.063) (Table 6).

There was no significant difference in daily level of PA of students of different year of study (χ^2^ = 3.475, *p* = 0.324, fi = 0.038), BMI (χ^2^ = 14.153, *p* = 0.117, fi = 0.044), alcohol consumption (χ^2^ = 19.81, *p* = 0.071, fi = 0.052), type of settlement (χ^2^ = 1.491, *p* = 0.684, fi = 0.025) and student smoking status (χ^2^ = 5.684, *p* = 0.128, fi = 0.048) (Table 6).

The model of binary logistic regression analysis showed that gender, presence of overweight or obesity and household income were significantly associated with students PA (Table 7). The odds of having regular daily PA for male students were 1.482 times higher than a female students (95% CI:1.178–1.865; *p* = 0.001). The odds of having regular daily PA for overweight or obese students were 0.732 times lower than a underweight and normal weight students (95% CI:0.578–0.928; *p* = 0.010). The odds of having regular daily PA for students whose household income was above average were 1.505 times higher than students whose household income was below average and average (95% CI:1.247–1.816; *p* < 0.001). Year of study, alchohol consumption, type of settlement and student smoking status were not significant predictors of students’ PA status.

## 4. Discussion

Our study shows that the students of the Faculty of Pharmacy of the University of Mostar, Bosnia and Herzegovina, had the highest percentage of engagement in some of regular PA, while the students of the Faculty of Pharmacy and Biochemistry of the University of Zagreb, Republic of Croatia, most often did not engage in regular PA, comparing to the students of other faculties. Likus et al. [27] in a study conducted in Poland indicate that most medical students do not engage in any form of PA, stating the lack of time due to faculty obligations to be the main excuse to engage in PA. According to results of the study by Dąbrowska-Galas et al. [28], which was also conducted in Poland, more than 80% of medical students engage in regular PA. The same study shows that university schedule, availability of sports centers and increased knowledge of health benefits have been associated with regular PA [28]. Another study conducted in Poland which was done by Ilow et al. [29] indicates low level of PA of pharmacy students. The authors point out that the most frequent barriers to PA are time limitation due to a busy study schedule [29]. Also, Ilow et al. [29] emphasize that PA should be promoted among students because of its positive influence on body weight and blood pressure. Martinović et al. [30] in a study conducted among biomedical students from Split (Republic of Croatia) show that more than a half of biomedical students has some kind of regular PA. The same study indicates that higher level of PA of biomedical students is associated with higher knowledge and positive opinions towards healthy lifestyle [30]. The authors point out that one of the major reasons for PA in a past few decades is predominantly the advancement in aesthetics and physical appearance as a way to achieve image of the perfect male and female bodies [30]. Martinović et al. [30] state that this could potentially be perceived as a risk, because the main reason for exercises should be maintaining good health, and that the influence of media and social networks could contribute to unreal body image aspirations.

The results of our research show that, only at the Faculty of Medicine Novi Sad of the University of Novi Sad, Republic of Serbia, there existed significant difference in daily level of PA between male and female medical students, where female medical students more frequently did not engage in regular PA in comparison to male medical students, while male medical students more frequently engaged in PA more than 1 h a day compared to female medical students. Having taken into consideration all medical students, regardless of the attended faculty, the obtained results of our research also indicate that female students compared to male students more frequently did not engage in regular PA. Male medical students more often did perform PA up to 30 min and more then 1 h per day in comparison with female medical students, while the female medical students more frequently had PA up to 1 h a day compared to male students. A study by Bin Abdulrahman et al. [22] conducted at medical colleges in Saudi Arabia to investigate medical students’ lifestyle habits, including PA shows that the greatest number of medical students exhibited healthy lifestyles to some extent. The same study indicates that these health-promoting behaviors differed by medical students’ gender, especially when speaking of PA and eating styles [22]. According to the results of the study conducted in Saudi Arabia, female medical students more often do not have any kind of PA compared to male medical students [22]. The study by Bin Abdulrahman et al. [22] also shows that male medical students engage in exercise more often than female medical students. The authors state that diminished levels of PA are in connection with an increase in the prevalence of diet-related non-communicable diseases, an overweight condition, and obesity among the young people [31,32]. The two main barriers for PA identified by medical students are lack of time and stress [23,33]. Jaremkówet al. [24] concucted a study among medical and dentistry students in Poland and indicate that, similar to our results, male students spent more time on both PA/exercise significantly more often than female students. In a study conducted among health science students in Spain, Romero-Blanco et al. [25] evaluated the level of PA of male and female students before and during the coronavirus lockdown, and showed that female students have a significant increase levels of PA in relation to male students. Romero-Blanco et al. [25] hold the opinion that the major reason for this phenomenon is the female students’ stronger motivation to reduce body weight gained during the coronavirus lockdown. The authors point out that perhaps male and female students may have different motivations and that the environmental factors influence one gender more strongly [25]. The results of the studies on motives for PA by gender indicate that some variables that motivated male, but not female students, are factors related to the environment (e.g., competition or social recognition), while the main motivation for female students is weight control [34]. Similar to the results of our research, the research by Stanford et al. [35] performed in the USA shows that male attending physicians, resident and fellow physicians and medical students are more likely to have regular PA than female students. However, a study by Blake et al. [36], conducted in United Kingdom (UK) on medical and nursing students in Canada, investigates predictors of PA level and provides evidence that gender was not a positive predictor of regular PA.

The obtained results of our research show significant difference in medical students’ daily level of PA regarding the year of study at the Faculty of Medicine of the University of Ljubljana, Republic of Slovenia, and the Faculty of Medicine of the University of Zenica, Bosnia and Herzegovina. At the Faculty of Medicine of the University of Ljubljana, Republic of Slovenia, there 4–6-year students in much higher percentage did not engage in regular PA and had PA up to 30 min a day in comparison to 1–3-year students. As for the Faculty of Medicine of the University of Zenica, Bosnia and Herzegovina, 1–3-year students more frequently did not engage in regular PA than 4–6-year students. Considering whole sample, regardless of attended faculty, the result of our research indicates that no significant difference was found in daily level of PA of medical students of different years of study. The results of a study by Romero-Blanco et al. [25] conducted in Spain among students of health sciences show that students in higher years of study have a lower level of PA than students in younger years of study. The authors emphasize that the main reason is the increasing level of obligations during the years of study [25]. A study done by Luciano et al. [37] among medical students in Italy indicates that there is no significant difference in the level of PA between students of younger and higher years of study. Luciano et al. [37] point out that improving PA and sleep as well as reducing sedentary lifestyle, would add benefit for the health of many students of medicine. The authors emphasize that medical school programs provide limited education on sleep and PA at the moment [38]. This is mainly because of the lack of qualified staff and dedicated time, and regarding such education as low priority [38].

Our results show that medical students whose household income was above average were the ones who significantly more often engaged in regular PA. They also had higher level of PA in relation to medical students whose household income was average and below average. The reason for this can potentially be the fact that the universities of the Western Balkans do not have programmes that would enable free sports centers for all students. Medical students are obliged to finance sports activities on their own, so those with higher household incomes have more opportunities to exercise in places such as gym, pool etc. Contrary to our results, the results of a study by Rejali et al. [39] conducted among medical and public health students in Iran indicate that students of lower socioeconomic status have a higher level of PA than students of higher socioeconomic status. The authors point out that organizing PA education programs, trainings and providing proving educational content in university health courses in order to encourage students for PA [39]. A study by the Awadalla et al. [40] conducted in Saudi Arabia shows that being a student in the college of medicine is associated with a high risk of physical inactivity, but, contrary to the results of our study, family income is not a significant predictor of physical inactivity. The obtained result of our research shows the odds of having regular daily PA were 1.505 times higher for medical students whose household income was above average in comparison to medical students whose household income was below average and average. As for socioeconomic standard and family income, there is a controversy about their influence on PA level [40]. While some studies reported that sedentary behavior and low levels of PA are associated with low socioeconomic status [41], other studies found out that high physical inactivity is associated with high socioeconomic status [42].

Similar to our research, the research by Zeńczak-Praga et al. [43], conducted on medical and physiotherapy students from Germany and Spain, shows that there is no significant association between the level of PA and BMI among students. Bergier et al. [44] in a study conducted among students including medical students from Ukraine and from the Visegrad countries (Hungary, Slovakia, Czech Republic and Poland), indicate that underweight and normal weight students from Visegrad countries have significantly higher amount of PA compared to Ukrainian students. Overweight and obese students have lower levels of PA than underweight and normal weight ones, but no significant difference is found between Visegrad and Ukrainian students [44]. The authors describe the results with advantageous economic situation of the students in the Visegrad countries that provides more free time that can be spent in increased PA [44]. Also, possible higher awareness of the PA role in a healthy lifestyle is proposed [44]. Pavičić-Žeželj et al. [45] in a study conducted among students of the Faculty of Medicine of the University of Rijeka (Republic of Croatia) point out that students with normal BMI are usually more physically active and that the desire for maintaining good health could contribute to a higher motivation to maintain a healthy lifestyle. A study by Catovic and Halilovic [46] conducted among medical students from the Faculty of Medicine of University of Sarajevo (Bosnia and Herzegovina) shows that there is a positive association between obesity and the average time spent in vigorous PA. Catovic and Halilovic [46] highlight that it is important to raise awareness about higher energy intake compared to energy consumption that may lead to the increase in body weight, and that PA is necessary to maintain a normal body mass and sustain good health.

Contrary to our results, in a study conducted among health science students in Spain, Romero-Blanco et al. [25], indicate a positive association of different alcohol consumption and the level of PA. Students who consume alcohol to a greater extent have a higher level of PA [25]. In a study conducted on medical students in Romania, Nasui et al. [47] show the differences between drinkers regarding the level of PA. The result of a study conducted in Romania indicates that medical students who have higher levels of PA are more prone to drinking alcohol in higher amounts [47]. The autors point out that regular PA is one of those lifestyle factors that may help individuals deal with stress [48,49]. Regular PA may function as an alternative to drinking [48,49].

According to the results obtained by Trivedi et al. [50], examined differences in obesity-related behaviors across rural-urban adult populations (aged 20 years or more) in the USA, in comparison to their urban counterparts, rural residents are more likely to be physically inactive, which is not in agreement with the results of our study, stating that there was no significant difference in daily level of PA of medical students of different type of dwelling settlement. The same study also shows that rural residents are more likely to report no PA at all [50]. The authors emphasize that when compared to their urban counterparts, rural residents have a higher representation of individuals less-educated about the significance of regular PA as modifiable risk factor for obesity among high-risk populations such as adult population in rural America [50]. It is highlight that techniques that entitle rural settlements to increase the availability of recreational resources and quality food markets need to be identified and disseminated more [50].

A study by Mansouri et al. [51] conducted among Iranian university students indicates a significant inverse association between PA and smoking, which is inconsistent with the results of our study that show no significant difference between the level of PA and the medical students’ smoking status. The authors state that it appears that PA alleviates the psychological distress associated with cigarettes smoking [52,53,54]. Furthermore, PA fills the students’ leisure time that may otherwise be spent on smoking [51]. The results obtained in a study by Tien Nam et al. [55] conducted in Vietnam on health science students indicate that a vigorous level of PA is associated with smoking. There may exist an explanation that in study which is conducted in Vietnam, health science students who are physically active are more prone to interact socially (students communicate with close friends who are smokers) and, as a result of that, smoke more [55].

The result of our research shows that the odds of having regular daily PA were 0.732 times lower for overweight or obese medical students compared to underweight and normal weight medical students. Contrary to our result, a study conducted by Gallo et al. [56] in Australia indicates that biomedical students with higher BMI are more likely to have increasing vigorous PA. Gallo et al. [56] state that, as expected, in a study conducted in Australia higher levels of vigorous PA of biomedical students are associated with modest reductions in percentage of body fat and blood glucose levels, confirming the significance of high-intensity exercise in the maintenance of metabolic health. The research by Medagama et al. [57] conducted among medical students in Sri Lanka shows that nutritional status do not represent a significant predictor of the student’level of PA. However, a study by Medagama et al. [57] indicates that overweight students are engaged in PA in a higher percentage than normal weight students.

Similar to our research, Blake et al. [36] in their study conducted among medical and nursing students in Canada indicate that the year of study does not represent a significant predictor of the their PA status. However, contrary to our results, the study by Medagama et al. [57] conducted on medical students in Sri Lanka, shows physical inactivity to be significantly associated with the year of study. The same study indicates that medical students in higher years of study are more likely to have PA compared to medical students at lower years of study [57]. The authors point out that greater academic pressure during the lower (pre-clinical) years of study than higher (clinical) may represent possible reason for these results [57].

Nasui et al. [47] in their study conducted on Romanian university medical students point out that there is a positive association of consuming alcohol in larger quantities and higher levels of PA, which is not in accordance with the result of our study that shows that alchohol consumption was not significant predictor of the PA status of medical students. The results of a study conducted by Nasui et al. [47] indicate that it seems to be probably that alcohol consumption plays a remarkable part in the social life of Romanian university medical students. The same study also shows that there is an increase in alcohol consumption during the academic years of Romanian medical students [47]. The authors state that regular PA may help individuals cope with stress during studies, but also other unhealthy habits, such as drinking or smoking can do the same [47].

The results of research by Trivedi et al. [50] conducted in the USA indicate that it is more likely for adults living in urban areas to meet sufficient level of PA compared to the ones coming from rural areas, which is not consistent with our results that did not show that dwelling settlement was a significant predictor of the level of PA of medical students. The reason for that is explained by higher percentage of individuals less-educated about the importance of PA among rural residents in comparison to urban residents [50].

According to Mansouri et al. [51] study conducted in Iran, PA is significantly associated with smoking. Iranian university students who are smokers are more likely to be physically inactive compared to the non-smoking students [51]. However, in a study conducted among medical students in Saudi Arabia, Torchyan et al. [58] indicate that highly physically active students are more likely to become smokers. The authors point out that students, owing to sports, during sports activities have more social interaction with their peers, which can be in connection with peer presure to start smoking [58]. The results of the study by Mansouri et al. [51] and study by Torchyan et al. [58] do not correspond with the results of our research that show that there was not any significant medical students’ smoking status influence on their level of PA.

A study by Kim et al. [59] conducted among health college students in South Korea, and the study by Kosendiak et al. [60] conducted among medical students in Poland indicate that there is a significant decrease in PA during COVID-19 pandemic and after pandemic measures have been reduced in relation to time before the beginning of the pandemic. Our study was conducted before the pandemic and future repeated research on the Western Balkan medical student population could have possible benefit because we would be able to establish if changes in PA levels were made due to changes in lifestyle of student population in past two years. This study also gives a basis for future research that can be conducted in order to determine the impact of PA promotion programs or university education between different Western Balkan countries on the prevalence of PA in the student population.

The importance of our research is that a broad study was conducted, for the first time in the Western Balkans, determining factors that affect the level of PA in the medical student population in this culturally specific region. Medical students, as future health workers, are key subjects in health promotion; they can take essential, necessary and continuous public health actions with the aim to improve life habits in the health studies student population. The contribution of this study implies application of its results to promote (national or international) public health activities specifically for future health workers, as well as for the overall student population.

Our study has several limitations. The research was conducted in the form of a cross-sectional study, which is a snapshot of the current situation. We were unable to observe changes over time and, consequently, the conclusions about the cause-effect relationship could not be made [61]. The online, anonymously filled-in self-administered questionnaire was used as an instrument for data collection, which is another limitation of our research. Despite emphasizing the anonymity of the survey and the confidentiality of the research results, respondents are frequently dishonest in giving answers [61], therefore the reliability of the answers cannot be established. Finally, a limitation of this study is the fact that it was conducted among students of medical faculties selected using convenience sampling method. Since the faculties were not randomly selected, the obtained results cannot be generalized to all students of medical faculties in the Western Balkans. For us, it was not possible to give precise numbers (response rate) of students from each faculty at the period of study performing, but the percentage of included students of all individual faculties definitly surpasses a representative 10% [62].

## 5. Conclusions

There are significantly more medical students who engaged than those who do not engage in some type of regular (daily) PA. Gender, overweight or obesity, and household income are significantly associated with medical students’ PA. Medical students who are more often involved in regular daily PA and have higher daily PA levels are more likely to be males whose household income is above average.

In order to improve the health of the student population, the educational institutions such as faculties, and public health authorities need to continuously investigate the PA of students, and introduce appropriate activities to increase their level of PA.

## Figures and Tables

**Table 1:** The treatable and preventable mortality rate and the percentage of the population that does not spend time on aerobic physical activity that improves health (unrelated to work) in relation to gender.

Country	Gender	Treatable and Preventable Mortality of Residents’ Rate (Deaths per 100,000 Inhabitants)	Reference	Percentage of Population that Spends no Time on Health-Enhancing (Non-Work-Related) Aerobic Physical Activity	Reference
Republic of Slovenia *	Male	345.97	[16]	44.4	[17]
	Female	148.72		52.1	
Republic of Croatia *	Male	527.95	[16]	60.0	[17]
	Female	214.83		65.3	
Bosnia and Herzegovina	Male	384.7 ***	[18]	71.3 ****	[18]
	Female	371.3 ***		79.7 ****	
Republic of North Macedonia **	Male	941.54 *****	[19]	Not available.	
	Female	897.1 *****			
Republic of Serbia **	Male	538.19	[16]	62.7	[17]
	Female	279.07		74.4	
European Union	Male	345.12	[16]	43.7	[17]
	Female	169.56		50.6	

* EU member; ** EU candidate for membership; *** expressed as the total mortality rate for the 5 leading diseases; **** expressed as total physical activity; ***** expressed as the total mortality rate for the 10 leading diseases.

**Table 2:** Distribution of medical students (*n* = 2452) by gender in relation to country.

Country	Gender				Total	
	Male		Female			
	*n*	%	*n*	%	*n*	%
Republic of Slovenia	27	12.4	191	87.6	218	8.9
Republic of Croatia	48	11.5	369	88.5	417	17.0
Bosnia and Herzegovina	179	20.4	699	79.6	878	35.8
Republic of North Macedonia	73	23.9	233	76.1	306	12.5
Republic of Serbia	110	17.4	523	82.6	633	25.8

**Table 3:** Daily level of physical activity of medical students from different medical faculties.

Country	Faculty	Daily Level of Physical Activity								*p* *
		I Do Not Engage in Regular Physical Activity		Up to 30 min a Day		Up to 1 h a Day		More than 1 h a Day		
		*n*	%	*n*	%	*n*	%	*n*	%	
Republic of Slovenia	Faculty of Medicine of the University of Ljubljana	104	47.7	37	17.0	32	14.7	45	20.6	<0.001
Republic of Croatia	Faculty of Pharmacy and Biochemistry of the University of Zagreb	138	50.9	46	17.0	36	13.3	51	18.8	
	Faculty of Medicine of the University of Rijeka	61	41.8	15	10.3	40	27.4	30	20.5	
Bosnia and Herzegovina	Faculty of Medicine of the University of Sarajevo	54	42.2	28	21.9	15	11.7	31	24.2	
	Faculty of Pharmacy of the University of Sarajevo	57	47.1	22	18.2	17	14.0	25	20.7	
	Faculty of Health Studies of the University of Sarajevo	62	29.8	45	21.6	42	20.2	59	28.4	
	Faculty of Medicine of the University of Zenica	50	24.4	61	29.8	60	29.3	34	16.6	
	Faculty of Pharmacy of the University of Mostar	25	22.7	24	21.8	29	26.4	32	29.1	
	Faculty of Health Studies of the University of Mostar	38	35.8	23	21.7	21	19.8	24	22.6	
Republic of North Macedonia	Faculty of Medicine of the University “St. Cyril and Methodius” Skopje	58	32.4	48	26.8	45	25.1	28	15.6	
	Faculty of Pharmacy of the University “St. Cyril and Methodius” Skopje	39	30.7	27	21.3	27	21.3	34	26.8	
Republic of Serbia	Faculty of Pharmacy of the University of Belgrade	56	45.2	18	14.5	29	23.4	21	16.9	
	Faculty of Medicine Novi Sad of the University of Novi Sad	140	37.8	69	18.6	75	20.3	86	23.2	
	Faculty of Pharmacy of the University of Business Academy Novi Sad	43	30.9	21	15.1	38	27.3	37	26.6	

* *p* value calculated by using χ^2^ test for categorical variables. Significant at *p* < 0.05. Note: Categories “up to 30 min/day” and “up to 1 h/day” do not overlap.

**Table 4:** Daily level of physical activity of medical students by gender in relation to the attended faculty.

Country	Faculty	Gender	Daily Level of Physical Activity								*p* *
			I Do Not Engage in Regular Physical Activity		Up to 30 min a Day		Up to 1 h a Day		More than 1 h a Day		
			*n*	%	*n*	%	*n*	%	*n*	%	
Republic of Slovenia	Faculty of Medicine of the University of Ljubljana	Male	9	33.3	5	18.5	5	18.5	8	29.6	0.408
		Female	95	49.7	32	16.8	27	14.1	37	19.4	
Republic of Croatia	Faculty of Pharmacy and Biochemistry of the University of Zagreb	Male	16	45.7	11	31.4	2	5.7	6	17.1	0.073
		Female	122	51.7	35	14.8	34	14.4	45	19.1	
	Faculty of Medicine of the University of Rijeka	Male	6	46.2	0	0.0	3	23.1	4	30.8	0.502
		Female	55	41.4	15	11.3	37	27.8	26	19.5	
Bosnia and Herzegovina	Faculty of Medicine of the University of Sarajevo	Male	9	40.9	4	18.2	3	13.6	6	27.3	0.948
		Female	45	42.5	24	22.6	12	11.3	25	23.6	
	Faculty of Pharmacy of the University of Sarajevo	Male	14	53.8	5	19.2	2	7.7	5	19.2	0.723
		Female	43	45.3	17	17.9	15	15.8	20	21.1	
	Faculty of Health Studies of the University of Sarajevo	Male	8	27.6	5	17.2	4	13.8	12	41.4	0.384
		Female	54	30.2	40	22.3	38	21.2	47	26.3	
	Faculty of Medicine of the University of Zenica	Male	12	22.2	11	20.4	17	31.5	14	25.9	0.098
		Female	38	25.2	50	33.1	43	28.5	20	13.2	
	Faculty of Pharmacy of the University of Mostar	Male	3	14.3	4	19.0	7	33.3	7	33.3	0.672
		Female	22	24.7	20	22.5	22	24.7	25	28.1	
	Faculty of Health Studies of the University of Mostar	Male	10	37.0	2	7.4	8	29.6	7	25.9	0.147
		Female	28	35.4	21	26.6	13	16.5	17	21.5	
Republic of North Macedonia	Faculty of Medicine of the University “St. Cyril and Methodius” Skopje	Male	11	23.9	19	41.3	8	17.4	8	17.4	0.05
		Female	47	35.3	29	21.8	37	27.8	20	15.0	
	Faculty of Pharmacy of the University “St. Cyril and Methodius” Skopje	Male	8	29.6	6	22.2	6	22.2	7	25.9	0.997
		Female	31	31.0	21	21.0	21	21.0	27	27.0	
Republic of Serbia	Faculty of Pharmacy of the University of Belgrade	Male	4	33.3	3	25.0	1	8.3	4	33.3	0.186
		Female	52	46.4	15	13.4	28	25.0	17	15.2	
	Faculty of Medicine Novi Sad of the University of Novi Sad	Male	21	28.4	13	17.6	14	18.9	26	35.1	0.047
		Female	119	40.2	56	18.9	61	20.6	60	20.3	
	Faculty of Pharmacy of the University of Business Academy Novi Sad	Male	6	25.0	4	16.7	7	29.2	7	29.2	0.923
		Female	37	32.2	17	14.8	31	27.0	30	26.1	

* *p* value calculated by using χ^2^ test for categorical variables. Significant at *p* < 0.05. Note: Categories “up to 30 min/day” and “up to 1 h/day” do not overlap.

**Table 5:** Daily level of physical activity of medical students by year of study in relation to the attended faculty.

Country	Faculty	Year of Study	Daily Level of Physical Activity								*p* *
			I Do Not Engage in Regular Physical Activity		Up to 30 min a Day		Up to 1 h a Day		More than 1 h a Day		
			*n*	%	*n*	%	*n*	%	*n*	%	
Republic of Slovenia	Faculty of Medicine of the University of Ljubljana	1–3	49	43.0	15	13.2	18	15.8	32	28.1	0.021
		4–6	55	52.9	22	21.2	14	13.5	13	12.5	
Republic of Croatia	Faculty of Pharmacy and Biochemistry of the University of Zagreb	1–3	87	50.6	31	18.0	24	14.0	30	17.4	0.813
		4–6	51	51.5	15	15.2	12	12.1	21	21.2	
	Faculty of Medicine of the University of Rijeka	1–3	41	46.6	10	11.4	18	20.5	19	21.6	0.139
		4–6	20	34.5	5	8.6	22	37.9	11	19.0	
Bosnia and Herzegovina	Faculty of Medicine of the University of Sarajevo	1–3	36	45.0	16	20.0	9	11.3	19	23.8	0.849
		4–6	18	37.5	12	25.0	6	12.5	12	25.0	
	Faculty of Pharmacy of the University of Sarajevo	1–3	32	43.2	14	18.9	14	18.9	14	18.9	0.245
		4–6	25	53.2	8	17.0	3	6.4	11	23.4	
	Faculty of Health Studies of the University of Sarajevo	1–3	52	30.8	37	21.9	32	18.9	48	28.4	0.798
		4–6	10	25.6	8	20.5	10	25.6	11	28.2	
	Faculty of Medicine of the University of Zenica	1–3	34	31.5	29	26.9	24	22.2	21	19.4	0.016
		4–6	16	16.5	32	33.0	36	37.1	13	13.4	
	Faculty of Pharmacy of the University of Mostar	1–3	17	22.4	17	22.4	19	25.0	23	30.3	0.953
		4–6	8	23.5	7	20.6	10	29.4	9	26.5	
	Faculty of Health Studies of the University of Mostar	1–3	37	37.0	20	20.0	21	21.0	22	22.0	0.205
		4–6	1	16.7	3	50.0	0	0.0	2	33.3	
Republic of North Macedonia	Faculty of Medicine of the University “St. Cyril and Methodius” Skopje	1–3	35	32.1	29	26.6	30	27.5	15	13.8	0.736
		4–6	23	32.9	19	27.1	15	21.4	13	18.6	
	Faculty of Pharmacy of the University “St. Cyril and Methodius” Skopje	1–3	27	29.0	21	22.6	21	22.6	24	25.8	0.799
		4–6	12	35.3	6	17.6	6	17.6	10	29.4	
Republic of Serbia	Faculty of Pharmacy of the University of Belgrade	1–3	43	50.6	11	12.9	17	20.0	14	16.5	0.310
		4–6	13	33.3	7	17.9	12	30.8	7	17.9	
	Faculty of Medicine Novi Sad of the University of Novi Sad	1–3	100	38.3	49	18.8	49	18.8	63	24.1	0.719
		4–6	40	36.7	20	18.3	26	23.9	23	21.1	
	Faculty of Pharmacy of the University of Business Academy Novi Sad	1–3	36	33.0	15	13.8	31	28.4	27	24.8	0.533
		4–6	7	23.3	6	20.0	7	23.3	10	33.3	

* *p* value calculated by using χ^2^ test for categorical variables. Significant at *p* < 0.05. Note: Categories “up to 30 min/day”and “up to 1 h/day” do not overlap.

**Table 6:** Daily level of physical activity according to gender, year of study, body mass index (BMI), alcohol consumption, household income, type of settlement and student smoking status.

	Variables	Daily Level of Physical Activity								*p* *
		I Do Not Engage in Regular Physical Activity		Up to 30 min a Day		Up to 1 h a Day		More than 1 h a Day		
		*n*	%	*n*	%	*n*	%	*n*	%	
Gender	Male	137	31.4	92	21.1	87	19.9	121	27.7	0.002
	Female	788	39.1	392	19.5	419	20.8	416	20.6	
Year of study	1–3	626	38.2	314	19.2	327	20.0	371	22.6	0.324
	4–6	299	36.7	170	20.9	179	22.0	166	20.4	
BMI	Underweight	73	42.9	28	16.5	36	21.2	33	19.4	0.117
	Normal weight	703	36.4	383	19.8	411	21.3	433	22.4	
	Overweight	118	40.0	64	21.7	53	18.0	60	20.3	
	Obese	31	54.4	9	15.8	6	10.5	11	19.3	
Alcohol consumption	I do not drink alcohol	318	38.7	128	15.6	196	23.8	180	21.9	0.071
	Occasionally	409	37.2	235	21.4	209	19.0	247	22.5	
	On weekends	158	36.5	100	23.1	86	19.9	89	20.6	
	Several times per week	35	41.2	19	22.4	13	15.3	18	21.2	
	Daily	5	41.7	2	16.7	2	16.7	3	25.0	
Household income	Below average	110	40.9	51	19.0	54	20.1	54	20.1	0.003
	Average	593	40.4	278	18.9	293	20.0	304	20.7	
	Above average	222	31.0	155	21.7	159	22.2	179	25.0	
Type of settlement	Rural	272	36.0	153	20.3	163	21.6	167	22.1	0.684
	Urban	653	38.5	331	19.5	343	20.2	370	21.8	
Smoking status	Non-smoker	707	37.0	392	20.5	403	21.1	409	21.4	0.128
	Smoker	218	40.3	92	17.0	103	19.0	128	23.7	

* *p* value calculated by using χ^2^ test for categorical variables. Significant at *p* < 0.05. Note: Categories “up to 30 min/day” and “up to 1 h/day” do not overlap.

**Table 7:** Association between independent variables and physical activity of medical students’.

Variables	B	S.E.	*p*	OR	95% CI for OR
Gender (male vs. female)	0.394	0.117	0.001	1.482	1.178–1.865
Year of study (1–3 vs. 4–6)	0.058	0.090	0.517	1.060	0.889–1.264
Overweight or obese (yes vs. no)	–0.312	0.121	0.010	0.732	0.578–0.928
Alcohol consumption (occasionally; on weekends; several times per week; and daily vs. I do not drink alcohol)	0.024	0.089	0.789	1.024	0.860–1.220
Household income (above average vs. below average and average)	0.409	0.096	<0.001	1.505	1.247–1.816
Type of settlement (urban vs. rural)	0.165	0.093	0.075	0.848	0.707–1.017
Student smoking status (smoker vs. non-smoker)	–0.145	0.101	0.151	0.865	0.709–1.054
Constant	0.477	0.104	<0.001	1.611	

Abbreviations: OR—odds ratio; CI—confidence interval; B—regression weight; S.E.—standard error.

## Data Availability

The data presented in this study are available on reasonable request from the corresponding author.
